# Influence of sitting behaviors on sleep disturbance and memory impairment in breast cancer survivors

**DOI:** 10.1002/cam4.3008

**Published:** 2020-03-23

**Authors:** Diane K. Ehlers, Jason Fanning, Alexis Sunderlage, Joan Severson, Arthur F. Kramer, Edward McAuley

**Affiliations:** ^1^ University of Nebraska Medical Center Omaha NE USA; ^2^ Wake Forest University Winston‐Salem NC USA; ^3^ University of South Carolina Columbia SC USA; ^4^ Digital Artefacts LLC Iowa City IA USA; ^5^ University of Illinois at Urbana‐Champaign Urbana IL USA; ^6^ Northeastern University Boston MA USA

**Keywords:** breast cancer, cognition, memory, physical activity, sedentary behavior, sleep

## Abstract

**Background:**

The purpose was to prospectively examine the effects of sedentary behaviors on subjective memory impairment in breast cancer survivors (BCS) and the extent to which sleep disturbances mediated this pathway.

**Methods:**

BCS (N = 380; *M*_age_ = 57.38 ± 9.25 years) completed questionnaires assessing demographics, health history, sitting behaviors, sleep disturbance, subjective memory impairment, and moderate‐to‐vigorous physical activity (MVPA) at baseline and 6‐month follow‐up. A subsample (N = 300) wore an accelerometer to objectively estimate sedentary time and MVPA. Structural equation modeling was used to test direct and indirect effects of self‐reported and objectively estimated sedentary behaviors on memory impairment (through sleep disturbance) across time. Models were adjusted for demographic, clinical, and MVPA covariates.

**Results:**

At baseline, more total daily sitting (*γ* = 0.23), occupational sitting (*γ* = 0.11), television viewing (*γ* = 0.15), and computer use (*γ* = 0.22) were associated with greater sleep disturbance, which was associated with greater memory impairment (*γ* = −0.22). Indirect effects of self‐reported sitting on memory were significant. At follow‐up, increased total daily sitting (*γ* = 0.08) and computer use (*γ* = 0.14) predicted increased sleep disturbance, which predicted increased memory impairment (*γ* = −0.09). The indirect path from increased computer use to memory impairment was significant (*β *= −0.01). In the accelerometer subsample, greater daily sedentary time at baseline was associated with less sleep disturbance (*γ* = −0.14) and memory impairment (indirect effect: *β *= 0.03).

**Conclusions:**

Findings provide early evidence that sedentary contexts may differentially influence sleep disturbance and memory impairment in BCS. Computer use and television viewing may pose the strongest risks to cognitive health. Disparate findings between objective and subjective sedentary measures warrant further research.

## Graphical abstract

Greater daily sitting among breast cancer survivors was associated with greater memory impairments, and this relationship was partially mediated by sleep disturbance. Screen‐based sitting behaviors may have the strongest influence on memory perceptions.

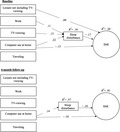

## 1 INTRODUCTION

Among breast cancer survivors (BCS), up to 75% report cognitive decline after diagnosis, with some reporting impairments up to 20 years after treatment ends.^1^, ^2^ Research suggests cancer‐related cognitive decline may represent an accelerated version of age‐related cognitive decline.^3^, ^4^ Indeed, studies have found that deficits among BCS may be 20%‐35% greater than women without cancer.^5^

The cognitive benefits of regular physical activity (PA) in older adults are well documented,^6^ and evidence in exercise oncology suggests PA may similarly benefit cancer survivors’ cognitive function.^7^, ^8^, ^9^, ^10^ Yet, recent research in aging has also indicated that moderate‐to‐vigorous PA (MVPA) consistent with public health recommendations may not be sufficient to offset the negative cognitive impacts of prolonged sedentary behavior.^11^, ^12^ Unfortunately, BCS spend as little as 2.6% of their day in MVPA, while sedentary behaviors comprise over two‐thirds of daily wake time.^13^ Studies in exercise oncology have linked sedentary behavior with greater fatigue, depression, pain, inflammation, and metabolic dysfunction,^14^, ^15^, ^16^ which are also thought to be underlying mechanisms of cancer‐related cognitive impairment (CRCI).^17^

In the general population, sitting behaviors have also been correlated with poorer sleep quality, and studies suggest the context in which sitting occurs may be more predictive of sleep outcomes compared with total sitting.^18^, ^19^ Vallance and colleagues^20^ observed increased odds of sleep disturbance among 1674 adults who watched greater than 6 hours of television (TV) per day. Although sleep disturbance is a commonly reported consequence of cancer,^21^, ^22^ and evidence supports MVPA for ameliorating sleep complaints in BCS,^23^ the influence of sedentary behaviors on sleep outcomes in cancer survivors has received little attention. Furthermore, sleep is a known correlate of cognitive function, particularly memory.^24^ Together, this evidence suggests sedentary behaviors may be linked with CRCI, and sleep quality may represent one mechanism explaining this relationship.

Using a prospective design, the present study examined the effects of daily sitting on subjective memory impairment (SMI) in BCS. We also investigated the extent to which self‐reported sleep disturbance mediated the pathway between daily sitting and SMI at baseline and across follow‐up (6 months). We hypothesized that greater total and screen‐based sitting (ie, TV viewing, computer use) would be associated with greater SMI, and effects would be indirect through sleep disturbance. We tested pathways of self‐reported sitting in the full sample (N = 380) and objectively estimated sedentary time in a subsample (n = 300).

## 2 MATERIALS AND METHODS

### 2.1 Participants and procedures

The present study employed a prospective, observational design. Study procedures have been previously published.^9^, ^25^, ^26^ Briefly, participants included 414 women aged 21 + years, diagnosed with breast cancer, who had completed primary treatment, and with access to an iPad. In the present analysis, we removed 33 participants due to missing (n = 32) or erroneous (n = 1) data on the sitting time questionnaire (final N = 380). Interested individuals enrolled in the study and completed measures via an iPad application (app). Participants completed the battery of assessments within 14 days of signing the consent form and were contacted 196 days (ie, 6 months) later to complete the assessments again. A subset agreed to wear an accelerometer, and those who provided complete accelerometer data at baseline (N = 300) were invited to wear the monitor again at follow‐up. All participants provided signed informed consent in accordance with the Institutional Review Board.

### 2.2 Measures

#### 2.2.1 Demographic and clinical information

Demographics and health history questionnaires were used to assess participant characteristics, breast cancer history, and general health history at baseline. Covariates included in data analyses were: age, education, menopausal status, months of adjuvant hormonal therapy, receipt of chemotherapy, and number of comorbidities.^9^

#### 2.2.2 Sedentary behavior

Sedentary behavior in the full sample was modeled as sitting time (minutes) on weekdays as assessed by the Sitting Time Questionnaire (STQ).^27^ The STQ measures self‐reported sitting while: (a) traveling to and from places (eg, work, shops); (b) at work; (c) watching TV; (d) using a computer at home; and (e) at leisure not including watching TV (eg, visiting friends, dining out). Sedentary behavior in the accelerometer subsample was estimated from a waist‐worn Actigraph GT3X accelerometer (Pensacola, FL) and modeled as average daily minutes sedentary using Freedson cutpoints.^28^ Individuals with at least 10 hours of wear time on at least 4 days were retained in analyses.^29^

#### 2.2.3 Sleep disturbance

The Pittsburgh Sleep Quality Index (PSQI)^30^ was used to assess sleep disturbance. The PSQI includes seven components comprising a global sleep score (range 0‐21). Higher scores indicate greater sleep disturbance.

#### 2.2.4 Subjective memory impairment

SMI was assessed using the Frequency of Forgetting scale (FOF).^31^ Respondents indicate how they feel about aspects of their memory ranging from 1 (very bad) to 7 (very good). The FOF includes four subscales: General Rating of Memory, Frequency of Forgetting, Frequency of Forgetting when Reading, and Remembering Past Events. Lower scores indicate greater SMI. For the present study, SMI was represented as a latent factor comprised of the subscales.

#### 2.2.5 Physical activity

The Godin Leisure‐Time Exercise Questionnaire (GLTEQ)^32^ and accelerometry were used to measure MVPA. The GLTEQ measures the frequency of mild, moderate, and strenuous exercise during an individual's leisure time over an average 7‐day period. Responses to the moderate and strenuous items were used to calculate an MVPA score. In the accelerometer subsample, MVPA was quantified using Freedson cutpoints^30^ and represented as average daily minutes of MVPA. Statistical models included baseline and follow‐up GLTEQ (full sample) or average daily MVPA (accelerometer subsample) as covariates to test the effects of sedentary behavior independent of MVPA.

### 2.3 Data analysis

The hypothesized pathway from sedentary behavior to SMI was tested using panel analyses within a covariance modeling framework. This approach allowed us to test relationships longitudinally while controlling for covariates and stability coefficients across time. Preliminary analyses indicated data were missing at random; therefore, the full information maximum likelihood estimation was used.^33^, ^34^ The extent of missing data ranged from 1.1% (FOF, GLTEQ) to 5.5% (PSQI) at baseline and 36.6% (GLTEQ) to 42.6% (STQ) at 6‐month follow‐up and were primarily the result of loss to follow‐up.

Prior to hypothesis testing, a confirmatory factor analysis (CFA) was conducted to model baseline and follow‐up SMI as latent factors. Because indicators were derived from the same measure, bivariate correlation analyses were conducted prior to the CFA to test the independence of each subscale. Correlations were moderate (*r* = 0.37‐0.63), with the exception of the correlation between General Rating of Memory and Frequency of Forgetting (*r* = 0.78 baseline, *r* = 0.80 follow‐up). As such, residual correlations of these indicators were included in the CFA (Figure S1). Hypothesized pathways were tested as follows: (a) direct effect of total weekday sitting on sleep disturbance, (b) direct effect of sleep disturbance on SMI, and (c) indirect effect of total sitting on SMI through sleep disturbance. To further test that the effect of sedentary behavior on SMI was indirect through sleep disturbance, we tested the direct effect of sedentary time on SMI. Next, we tested the effects of each STQ domain, while controlling for other domains, as described above. Finally, we tested the above models in the accelerometer subsample.

Baseline age, education level, menopausal status, months of hormonal therapy, receipt of chemotherapy, and comorbidities and MVPA as reported/measured baseline to follow‐up were included as covariates. Stability coefficients were calculated to account for correlation between baseline and follow‐up variables derived from the same measure. Variables were Winsorized at three standard deviations from the mean. Significant effects are presented as standardized effects at a one‐tailed alpha of *P *< .05. Covariate coefficients are not included in figures for clarity purposes but are reported in the text. Model fit was assessed using standard indices: nonsignificant normal theory weighted chi‐square (*χ* ^2^), comparative fit index (CFI > 0.90), root mean square error of approximation (RMSEA < 0.05), and standardized root mean residual (SRMR < 0.08).^35^

## 3 RESULTS

### 3.1 Full sample

Tables S1 and S2 describe the sample and summarize primary outcomes, respectively. The measurement model for the SMI latent factors provided excellent fit to the data (*χ* ^2^ = 13.46 [13], *P *= .41, CFI = 1.00, RMSEA = 0.009 [90% CI = 0.00 to 0.05], SRMR = 0.019), and all indicators loaded significantly on the hypothesized factors (all *P* < .001; Figure S1). The structural model testing the hypothesized pathway from total weekday sitting to SMI had excellent fit (*χ* ^2^ = 132.38 [112], *P* = .09, CFI = 0.99, RMSEA = 0.02 [90% CI = 0.00 to 0.036], SRMR = 0.046). The hypothesized model testing the effects of each STQ domain had good fit to the data. However, the model indicating significant direct effects of STQ domains on SMI was retained and had similarly good fit (*χ* ^2^ = 342.81 [240], *P* =< 0.001, CFI = 0.96, RMSEA = 0.03 [90% CI = 0.025 to 0.042], SRMR = 0.057). Stability coefficients were acceptable and ranged from 0.38 (STQ non‐TV leisure time) to 0.87 (memory latent factor).

#### 3.1.1 Baseline

**Figure 1:**
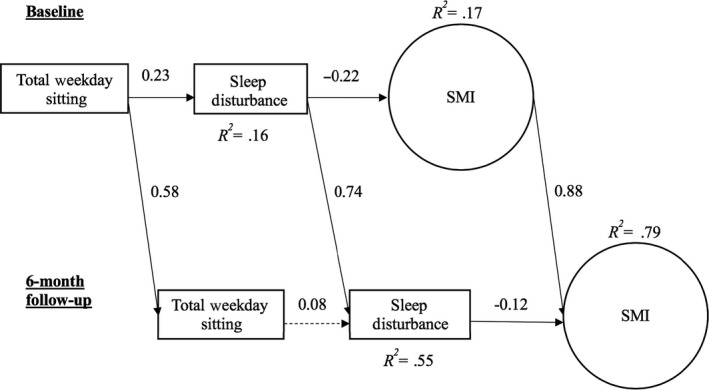
Panel model of effects of total weekday sitting time on sleep disturbance and memory impairment. Solid lines indicate significant paths. ^a^Positive path: more daily sitting associated with more sleep disturbance. ^b^Negative path: more sleep disturbance associated with more memory impairment

**Figure 2:**
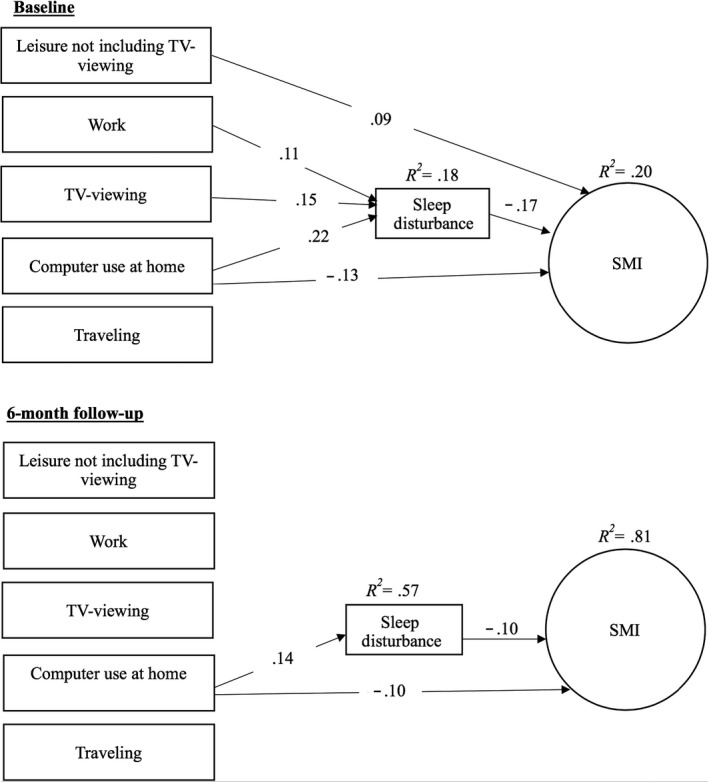
Panel model of effects of sitting domains on sleep disturbance and memory impairment. Solid lines indicate significant paths

A direct path from total weekday sitting to sleep disturbance was observed (*z* = 4.77, *P* < .001) whereby more weekday sitting was associated with more sleep disturbance (Figure 1). Direct paths to sleep disturbance from weekday sitting while at work, TV viewing, and using the computer at home were also observed (work: *z* = 1.95, *P* = .03; TV: *z* = 3.02, *P* = .002; computer: *z* = 4.47, *P* < .001). Specifically, more time spent sitting at work, while watching TV, and when using the computer were associated with more sleep disturbance (Figure 2). A direct path from sleep disturbance to SMI was observed in both models (*z *= −4.02, *P* < .001 [Figure 1]; *z *= −3.07, *P* = .001 [Figure 2]) in which greater sleep disturbance was associated with greater SMI. The indirect path from total sitting to SMI, through sleep disturbance, was significant (*β *= −0.05, *z *= −3.06, *P* = .001). Similarly, the indirect paths from TV viewing and computer use were significant (TV: *β *= −0.03, *z *= −2.14, *P* = .02; computer: *β *= −0.04, *z *= −2.51, *P* = .006), and the indirect path from sitting at work was marginally significant (*β *= −0.02, *z *= −1.64, *P* = .05). We also observed direct effects of leisure sitting (no TV) and computer use on SMI (leisure: *z* = 1.64, *P* = .05; computer: *z *= −2.39, *P* = .009). Greater leisure sitting was associated with less SMI, while greater computer use sitting was associated with greater SMI.

#### 3.1.2 Six‐month follow‐up

Increased total daily sitting was marginally associated with increased sleep disturbance (*z* = 1.61, *P* = .05; Figure 1). Increased home computer use was significantly associated with increased sleep disturbance (*z* = 2.85, *P* = .002), while increased leisure sitting (no TV) was marginally associated with decreased sleep disturbance (*z *= −1.59, *P* = .056; Figure 2). Increased sleep disturbance was associated with increased SMI (*z *= −2.04, *P* = .02). Only the indirect path from change in computer use to change in SMI was statistically significant (*β *= −0.01, *z *= −1.66, *P* < .05). Finally, a direct path from increased computer use to SMI was observed (*z *= −2.16, *P* = .02).

### 3.2 Accelerometer subsample

**Figure 3:**
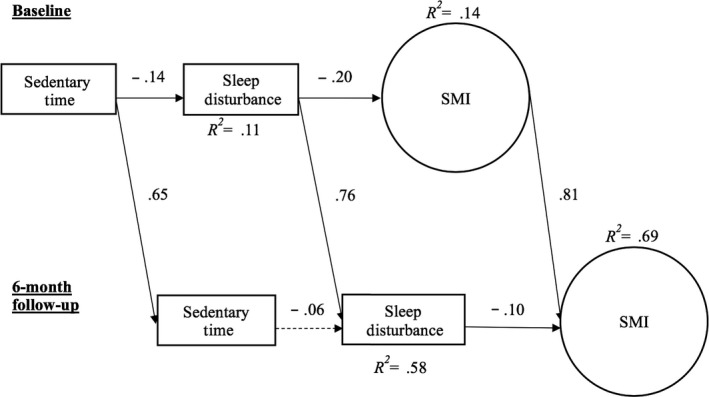
Panel model of effects of accelerometer‐estimated, average daily sedentary time on sleep disturbance and memory impairment. Solid lines indicate significant paths

The structural model testing the hypothesized pathway from average daily sedentary time to SMI had excellent fit to the data (*χ* ^2^ = 135.54 [114], *P* = .08, CFI = 0.988, RMSEA = 0.03 [90% CI = 0.00 to 0.04], SRMR = 0.05) (Figure 3).

#### 3.2.1 Baseline

A direct path from daily sedentary time to sleep disturbance was observed such that more daily sedentary behavior was associated with less sleep disturbance (*z *= −2.30, *P* = .01). Less sleep disturbance was, in turn, directly associated with less SMI (γ = −0.20, *z *= −3.342, *P* < .001). While the direct effect of sedentary time on SMI was not significant (*P* = .30), the indirect effect through sleep disturbance was significant (*β* = 0.03, *z* = 1.92, *P* = .03).

#### 3.2.2 Six‐month follow‐up

Change in objectively estimated sedentary behavior was not associated with the change in sleep disturbance (*P* = .12) or SMI (*P* = .46). A direct path from increased sleep disturbance to increased SMI was observed (*z*=−2.14, *P* = .02).

## 4 COVARIATES

Among covariates, age (*γ* = −0.16, *z *= −2.78, *P* = .003), bachelor's degree (*γ* = −0.11, *z *= −2.22, *P* = .01), hormonal therapy (*γ* = −0.14, *z *= −2.92, *P* = .002), and comorbidities (*γ* = 0.21, *z* = 4.06, *P* < .001) were associated with sleep disturbance. Coefficients indicated greater sleep disturbance among women who were younger, less educated, reported fewer months of hormonal therapy, and reported more comorbidities. Age, menopausal status, and comorbidities were directly associated with SMI (age: *γ* = 0.22, *z* = 3.62, *P* < .001; menopause: *γ* = −0.10, *z *= −1.70, *P* < .05; comorbid: *γ* = −0.22, *z *= −4.11, *P* < .001), with memory impairment being greater in women who were younger, postmenopausal, and who reported more comorbidities. Across follow‐up, having a bachelor's degree was significantly associated with increased sleep disturbance (*γ* = 0.13, *z* = 2.60, *P* = .005), while age was associated with decreased SMI (*γ* = −0.10, *z *= −1.81, *P* = .04).

## 5 DISCUSSION

This study provides evidence of associations among sedentary behavior, sleep disturbance, and SMI in BCS. While it is not clear if total daily sitting has meaningful effects on sleep disturbance and memory perceptions, findings suggest various types of sedentary behavior may have differential influence. Contradictory findings between self‐reported and accelerometer‐based analyses underscore the need for additional research focused on understanding the health impacts of behavioral patterns and contexts across the day. Analysis of sedentary contexts suggests screen time, especially time spent on the computer at home, may most contribute to the negative cognitive effects observed at baseline and across time. Findings partially support hypotheses from the aging literature linking sedentary behavior with cognition and brain health^12^, ^36^ and add to priority research areas in cancer survivorship.^37^

While several studies have documented deleterious effects of sedentary behaviors on physical and psychosocial functions in noncancer and cancer populations,^14^, ^15^, ^16^, ^20^, ^38^, ^39^, ^40^ our findings contribute to emerging evidence that these negative effects may extend to cognitive function. In a recent systematic review, Falck, Davis, and Liu‐Ambrose^36^ observed an association between increased sedentary behavior and reduced cognitive function in older adults. We observed similar relationships in which associations were stronger at baseline and weaker across time. This is not surprising given the observational study design and limited time to follow‐up in which significant lifestyle and cognitive changes are not likely to occur. Likewise, our findings partially corroborate those of Marinac and colleagues,^41^ who observed a significant inverse association between prolonged sitting, but not total sitting time, and objectively measured cognitive function in 30 BCS.

A strength of the present study is the use of both objective and subjective measures of sedentary behavior, as researchers warn that reliance upon total daily sedentary time as an independent variable may limit our understanding of sedentary behavior as a health risk factor.^42^ Despite this, the conflicting results between self‐reported and objectively estimated sedentary time warrant further investigation. While the role of total sitting time in our study and others remains unclear,^41^ results indicate that certain types of sitting may differentially influence SMI. TV viewing was indirectly associated with SMI at baseline, and computer use was directly and indirectly associated with SMI at baseline and across follow‐up. Several previous studies have demonstrated the health risks associated with excessive TV viewing,^43^, ^44^ including recent evidence linking hours of TV viewing with poor sleep quality, global cognition, and memory in middle‐aged and older adults.^11^, ^18^, ^36^ For example, a recent UK Biobank study demonstrated inverse associations between TV viewing and performance on fluid intelligence and short‐term memory tasks in adults aged 37‐73 years. However, contrary to our study, the authors observed an inverse association between sitting while traveling (operationalized as hours of driving) and cognition and a positive association between nonoccupational computer use and cognition.^11^ In studies of cancer survivors, TV viewing alone and screen time (combined TV + computer) have been associated with lower quality of life and poorer sleep outcomes.^20^, ^45^

Of further interest is the finding that leisure non‐TV sitting was directly associated with less memory impairment at baseline. This may also explain the equivocal findings related to total daily sedentary time and provides additional evidence that the limited metabolic costs of sitting may have a lesser influence on cognitive health compared with sitting contexts. Example behaviors included in the leisure, non‐TV sitting item include “visiting friends, movies, dining out, etc”^27^ Therefore, it is possible that this time was spent in activities such as reading and socializing, which are evidenced to be cognitively enriching.^46^ Further research to dissect the unique cognitive influences of physical activity, intellectual and social sedentary behavior, and screen‐based sedentary behavior is warranted. Additionally, the timing of sitting behaviors across the day was not discernable from our data. Previous evidence has linked TV viewing and computer use immediately before bed to circadian dysfunction and disrupted sleep, although most studies have focused on children and adolescents.^47^ Unfortunately, sleep disturbances are widespread among BCS, with prevalence estimated to be 40% according to a recent meta‐analysis.^48^ Given that sleep mediated the effects of screen‐based sitting on SMI, but not the effects of non‐TV sitting, future interventions may consider a multiple behavior approach aimed at reducing specific types of sedentary behavior associated with poor sleep hygiene. Mobile time‐use diaries, which have undergone feasibility testing in BCS,^49^ may provide the opportunity to better understand sedentary contexts, patterns, and timing, and identify points of intervention, specifically around screen‐based behaviors.

## 6 STRENGTHS AND LIMITATIONS

Strengths of the present study include recruitment of a national sample of BCS, prospective analysis of pathways, modeling of SMI as a latent variable, and inclusion of multiple measures of sedentary behavior. However, there are also limitations to be considered. While the prospective design is a strength, data are observational and prohibit causal interpretation. Findings warrant investigations in a randomized controlled trial to enhance our understanding of sedentary behaviors and CRCI. Additionally, SMI was self‐reported and is likely not comparable to objective cognitive performance.^5^ Nevertheless, perceptual beliefs about one's cognition are important and should be considered alongside cognitive performance. Finally, despite our large sample, participant attrition at follow‐up was substantial. Efforts to retain participants were made (ie, monetary incentives, push notifications, reminder emails). However, stronger efforts may be required to retain participants in technology‐based studies employing a no‐contact follow‐up.

## 7 CONCLUSIONS

Cancer‐related cognitive impairment continues to be a concern with no evidence‐based treatment. Our findings suggest there may be different effects of various sitting behaviors on cognitive function in BCS. Conflicting findings between the full and accelerometer samples emphasize the need for additional research to understand how the quantity vs type of sedentary behavior impact sleep and cognitive outcomes in BCS. Furthermore, while the present study suggests sleep disturbance may be one mechanism by which sedentary behaviors influence SMI in BCS, research in other populations provides insights into other mechanisms warranting investigation (eg, neural, cardiometabolic biomarkers).^12^, ^50^ As most of the evidence focused on sedentary behaviors and cognition has been derived in healthy populations, additional investigations in cancer populations are needed.

## CONFLICT OF INTEREST

None to declare.

## AUTHOR CONTRIBUTIONS

DKE was involved in conceptualization, data curation, analysis, funding, methodology, and writing–original draft. JF was involved in data analysis/interpretation and writing–review/editing. AS was involved in writing–original draft. JS was involved in methodology and project administration. AFK was involved in methodology, supervision, and writing–review/editing. EM was involved in conceptualization, funding, methodology, supervision, and writing–review/editing.

## Supporting information

Fig S1

Table S1

Table S2

## Data Availability

Data will be available without restriction by request to the corresponding author.
