# Supplementary material for: Influence of sitting behaviors on sleep disturbance and memory impairment in breast cancer survivors
Source: Cancer Med. 2020 Mar 23;9(10):3417–24. doi: 10.1002/cam4.3008 (PMC7221435; doi:10.1002/cam4.3008)
Supplement: Supplementary file 2 — Table S1 [file CAM4-9-3417-s002.docx]

|  |  | M±SD^a^ | |
| --- | --- | --- | --- |
|  |  | *n*(%) | |
| Age (years) | | 57.38 | ±9.52 |
| Caucasian | | 364 | ❨95.8❩ |
| Married | | 297 | (78.2) |
| Bachelor’s Degree | | 298 | ❨78.4❩ |
| Income > $75,000 per year | | 273 | ❨71.8❩ |
| Employment Status | |  |  |
| Full-time | | 144 | (37.9) |
| Part-time | | 49 | (12.9) |
| Retired | | 132 | (34.7) |
| Other | | 55 | (14.5) |
| Cancer Stage | | | |
|  | 0 | 33 | ❨8.7❩ |
|  | 1 | 154 | ❨40.5❩ |
|  | 2 | 126 | ❨33.2❩ |
|  | 3 | 60 | ❨15.8❩ |
|  | 4 | 7 | ❨1.8❩ |
| Months since diagnosis | | 93.12 | ±70.39 |
| Treatment History | |  |  |
| Chemotherapy | | 260 | ❨68.4❩ |
| Radiation | | 260 | (68.4) |
| Chemotherapy+Radiation | | 188 | (59.5) |
| Hormonal therapy (months) | | 20.07 | ±29.48 |
| Post-menopausal | | 342 | ❨90.0❩ |
| Number of comorbidities (out of 10)^b^ | | 0.98 | ±0.98 |
| 0 | | 142 | ❨37.4❩ |
| 1-2 | | 208 | ❨54.7❩ |
| >3 | | 30 | ❨7.9❩ |

^a^Mean, Standard Deviation

^b^Sum of: arthritis, osteoporosis, asthma, chronic obstructive pulmonary disorder (COPD), congestive heart failure, heart attack, stroke, dementia, Type 2 diabetes, depression
