# Supplementary material for: Influence of sitting behaviors on sleep disturbance and memory impairment in breast cancer survivors
Source: Cancer Med. 2020 Mar 23;9(10):3417–24. doi: 10.1002/cam4.3008 (PMC7221435; doi:10.1002/cam4.3008)
Supplement: Supplementary file 3 — Table S2 [file CAM4-9-3417-s003.docx]

|  | Baseline | Follow-up |
| --- | --- | --- |
|  | M±SD | M±SD |
| Weekday Sitting Time | 592.51±300.42 | 601.14±315.97 |
| Weekday Travel | 75.44±80.80 | 79.04±90.41 |
| Weekday Work | 174.03±196.21 | 173.49±188.24 |
| Weekday TV | 139.10±127.62 | 145.89±127.25 |
| Weekday Computer at Home | 118.90±104.09 | 117.66±111.24 |
| Weekday Leisure (non-TV) | 85.03±97.36 | 82.34±79.80 |
| Average Daily Sedentary Time (accelerometer) | 599.47±72.09 | 623.27±78.88 |
| General Rating of Memory | 4.66±1.46 | 4.89±1.36 |
| Frequency of Forgetting | 27.93±6.53 | 29.05±6.43 |
| Frequency of Forgetting When Reading | 11.20±2.77 | 11.46±2.67 |
| Remembering Past Events | 9.64±2.81 | 9.84±2.82 |
| Sleep Disturbance | 7.03±3.79 | 6.35±3.73 |
| Godin Weekly Leisure Activity Score (MVPA) | 26.32±19.63 | 26.42±19.19 |
| Average Daily MVPA (accelerometer) | 29.84±22.55 | 27.85±20.88 |
